# Acute-phase protein concentrations in serum of clinically healthy and diseased European bison (*Bison bonasus*) – preliminary study

**DOI:** 10.1186/s12917-021-03133-z

**Published:** 2022-01-10

**Authors:** Małgorzata Pomorska-Mól, Kacper Libera, Magdalena Larska, Michał K. Krzysiak

**Affiliations:** 1 Department of Preclinical Sciences and Infectious Diseases, Faculty of Veterinary Medicine and Animal Science, Poznań University of Life Sciences, ul. Wołyńska 35, 60-637 Poznań, Poland; 2 Department of Virology, National Veterinary Research Institute, Al. Partyzantów 57, 24-100 Puławy, Poland; 3 Białowieża National Park, Park Pałacowy 11, 17-230 Białowieża, Poland; 4 Institute of Forest Sciences, Faculty of Civil Engineering and Environmental Sciences, Bialystok University of Technology, Białystok, Poland

**Keywords:** European bison, Acute phase proteins, Wildlife management, Hp, SAA, AGP

## Abstract

**Background:**

This is the first report describing levels of APPs in European bison. Serum concentration of acute phase proteins (APPs) may be helpful to assess general health status in wildlife and potentially useful in selecting animals for elimination. Since there is a lack of literature data regarding concentration of APPs in European bisons, establishment of the reference values is also needed.

**Methods:**

A total of 87 European bison from Polish populations were divided into two groups: (1) healthy: immobilized for transportation, placing a telemetry collar and routine diagnostic purposes; and (2) selectively culled due to the poor health condition. The serum concentration of haptoglobin, serum amyloid A and α1-acid-glycoprotein were determined using commercial quantitative ELISA assays. Since none of the variables met the normality assumptions, non-parametric Mann-Whitney U test was used for all comparisons. Statistical significance was set at p < 0.05. Statistical analyses were performed using Statistica 13.3 (Tibco, USA).

**Results:**

The concentration of haptoglobin and serum amyloid A was significantly higher in animals culled (euthanised) due to the poor condition in respect to the clinically healthy European bison. The levels of α1-acid-glycoprotein did not show statistical difference between healthy and sick animals.

**Conclusions:**

Correlation between APPs concertation and health status was proven, therefore the determination of selected APPs may be considered in future as auxiliary predictive tool in assessing European bison health condition.

## Background

At the beginning of XX century, European bison *(Bison bonasus)* extinct from wild, when the last surviving animals were killed in the Białowieża Primeval Forest after World War I. Fortunately, the biggest wild herbivore in Europe have survived and now, 70 years after the first European bison had been released into the wild again, its world population is estimated at more than 9100 heads [1]. Unfortunately, growing numbers and density of the population exceeding capacity of the available habitats, increasing anthropopression and limited gene pool increase nowadays the risk of health threats such as exposure to infectious and invasive pathogens [2]. Therefore, it is crucial to support European bison population development by proper wildlife management. One of the tools that can be used to manage wildlife species is selective elimination (culling) of individuals, which genotype (and/or lineage) or health impairment make them undesirable in a herd. Apart from that, diseased individuals may threaten other animals in the population in case of contagious diseases, which, to make things worse, may also have zoonotic potential and be a challenge for public health [3, 4]. This applies especially to tuberculosis, which is currently a real zoonotic health hazard in wild ruminant populations including European bison [5]. The question which arises is how to accurately select individuals to be eliminated. The potential solution may be taken from farm animals medicine, in particular cattle, which is relatively closely related to European bison. It involves determining serum acute phase proteins (APPs) concentration, which reflects animal health status under different pathological conditions [6–8]. It could also be used as a predictive tool describing the chance of recovery based on the severity of the disease. Besides, determination of APPs can be applied as a reliable solution to assess general health status, which is crucial in case of endangered animal species, particularly with limited genes pool. The concentrations and type of APPs differ within the species [9, 10]. In cattle, the most important APPs are haptoglobin (Hp), serum amyloid A (SAA) and α1-acid-glycoprotein (AGP) [11], which levels are used as biomarkers of various pathological conditions and as predictive values for mastitis [12], respiratory diseases [13], and lameness [14]. However, no reports investigating concentration of APPs in European bison are available. Although, some studies have been described in other wildlife non-bovid species, nevertheless the data is very limited [15–17]. The secretion of APPs is associated with the acute phase reaction (APR) [18]. The APR is an innate response of the body to the disturbance of homeostasis which may be associated with various factors i.e. infection, damage of tissues, neoplastic hyperplasia, immunological disorders [19–21]. The goal of the acute phase response is to recover homeostasis and eliminate the causative factor. It comprises numerous hormonal, metabolic and neurological changes which occur within a short period of time after the injury, at the beginning of infection or inflammation [18]. Referring to cattle (as closely related to European bison domesticated species), the Hp, SAA and AGP have been described as the most significant markers of APR and are considered the major APPs used in the diagnostics. The level of Hp in cattle increases significantly in the course of APR (from nearly zero to about 2 g/l within 48 h from stimulation. The major biologic function of Hp is to bind haemoglobin in an equimolar ratio with very high affinity to prevent haemoglobin-mediated renal parenchymal injury and loss of iron following intravascular haemolysis [22]. Serum amyloid A seems also to be a good indicator of acute phase reaction in cattle. The synthesis of certain members of the family SAA proteins is significantly increased during inflammation [23]. SAA proteins can be considered as apolipoproteins because they associate with plasma lipoproteins mainly within the high-density range. Physiological role of SAA in the immune response during inflammation is not well understood, but various effects have been described. These include e.g., inhibition of lymphocyte proliferation, detoxification of endotoxin, inhibition of platelet aggregation, inhibition of thrombocytes aggregation, and inhibition of oxidative reaction in neutrophils [23]. Therefore, the aim of our preliminary study was to determine serum concentration of selected APPs (haptoglobin, serum amyloid A and α1-acid-glycoprotein) in clinically healthy European bison and animals eliminated under the clinical suspicion of different pathological conditions, followed by post-mortem evaluation [24]. We hypothesised that concentrations of selected APPs were higher in the eliminated European bison (ELIM) comparing to clinically healthy animals (HEAL).

## Methods

### Samples

Blood samples were collected from 87 European bison, including 40 samples from clinically healthy individuals (HEAL) and 47 samples from individuals eliminated due to different pathological conditions (ELIM). In HEAL, there were 27 females and 13 males in the average age of 6.17 (range: 1-16 years). While in ELIM there were 30 females and 17 males in the average age of 6.5 (range: 1-20 years). The clinically healthy European bison were pharmacologically immobilized for transportation, placing a telemetry collar, or routine diagnostic purposes according to the previously described protocols [25]. Briefly, the combination of xylazine and etorphine were used to immobilization. The preparations were administered with specialized pneumatic Dan-Inject applicators. Following a successful shot, the animal is immobilized within 15 min and thereafter veterinary and animal husbandry manipulations are possible. In order to awake the European bisons the mixture of atipamezole, naloxone and diprenorphine were used. The selected individuals were culled after the health assessment (ELIM) by the herd supervising veterinarian and under the decision of Ministry of Environment. Briefly, the health status assessment includes integumentary system examination, orifices inspection (check for any discharges), reproductive organs examination, sight organ examination as well as body condition assessment. The animals were sampled in accordance with the appropriate regulations and permits (Polish General Directorate for Environmental Protection: Regulations DOP-OZGIZ.6401.06.7.2012.1 s and DOPOZ. 6401.06.7.2012.1 s1.Warsaw, 2012; Polish General Directorate for Environmental Protection: Regulations DZPWG.6401.06.23.2014.km2.; and Polish Ministry of the Environment, Regulation: DLP-III-0771-5/42,173/14/ZK). The blood samples were taken at capture and collected from the jugular vein into sterile tubes with clot activator for serum separation. The poor quality samples were excluded prior to the study for ensuring the high reliability of results. Serum was harvested from the blood samples by centrifugation (3000 × g for 15 min at room temperature) and stored at -80° C for further analysis (not longer than one month).

### Determination of APPs

The concentrations of haptoglobin, serum amyloid A and α1-acid-glycoprotein in serum were measured using commercial ELISAs and colorimetric assays according to manufacturer’s guidelines, which included Haptoglobin Kit Phase Range and Phase Serum Amyloid A assays (Tridelta Development Ltd County Kildare, Ireland) and Cow α1-acid glycoprotein (AGP) ELISA (Life Diagnostic, West Chester, USA). Serum samples were tested in duplicate. Prior to AGP and SAA analyses samples were diluted as follows: 1:10,000 for AGP and 1:500 for SAA. The concentrations of the APP was calculated based on standard curve for each protein using the FindGraph computer software (UNIPHIZ Lab, Vancouver, Canada). All assays were preliminarily validated in our laboratory before being applied in European bison samples. For this purpose, precision, accuracy and limit of detection were calculated. Within-run coefficients of variation (CVs) were calculated after the analysis of 2 serum samples with low and high proteins concentrations eight times in a single run. Between-run CVs were obtained by measuring the same samples in eight separate runs carried out on three different days. All samples used were frozen in aliquots and only the vials needed for each run were used. Accuracy was investigated by linearity under dilution; in brief, two European bison serum samples were diluted (1:2; 1:4; 1:8; 1:16, 1:32) with sample diluents. The limit of detection was calculated as the lowest concentration of APP that could be distinguished from a zero sample, and was taken as the mean + 3 standard deviations (SD) of 12 replicates of blank sample, tested in one analytical run.

### Statistical analysis

In order to select proper statistical tools to evaluate the differences between healthy (HEAL) and European bison eliminated due to poor health conditions (sick animals) (ELIM), the normality of data was verified by using Shapiro-Wilk test. Since none of the variables met the normality assumptions, non-parametric Mann-Whitney U test was used for all comparisons. Statistical significance was set at p < 0.05. Statistical analyses were performed using Statistica 13.3 (Tibco, USA).

## Results

### Analytical validation of the assays

For Hp mean within-run and between-run CVs were 8.11% and 7.11%, respectively. Dilution studies resulted in linear regression equations with a correlation coefficient of 0.98 showing that the method measures the protein in a linear manner. The detection limit of the assay was 0.005 mg/ml.

For SAA mean within-run and between-run CVs were 5.15% and 5.35%, respectively. Dilution studies resulted in linear regression equations with a correlation coefficient of 0.99 showing that the method measures the protein in a linear manner. The detection limit of the assay was 3.49 µg/ml.

For AGP mean within-run and between-run CVs were 4.08% and 4.37%, respectively. Dilution studies resulted in linear regression equations with a correlation coefficient of 0.99 showing that the method measures the protein in a linear manner. The detection limit of the assay was 15.6 µg/ml.

### Acute phase protein concentrations

**Fig. 1:**
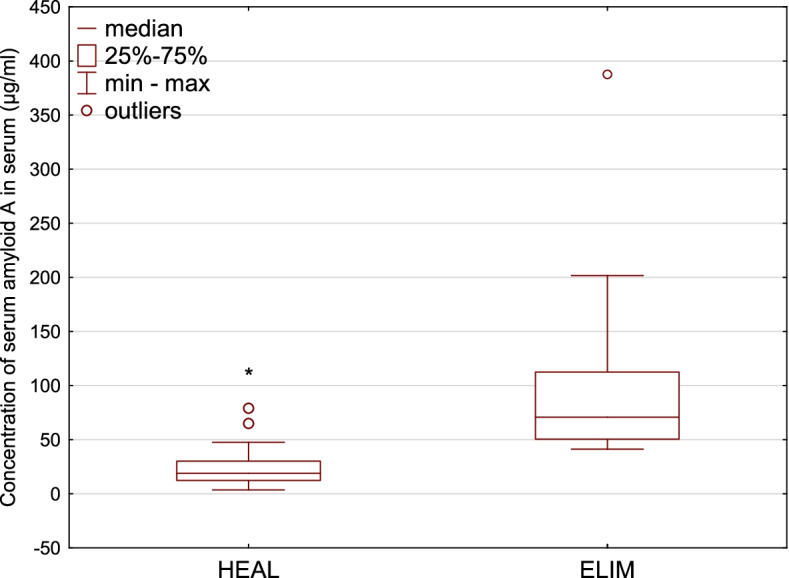
A boxplot for the concentration of serum amyloid A in serum of European bison. The line inside the box is the median. The top and bottom lines of the box are the first and third quartiles, respectively. The top and bottom whiskers are the minimum and maximum concentrations, circles represent outliers. * - statistically significant differences between HEAL and ELIM (*p *< 0.01)

**Fig. 2:**
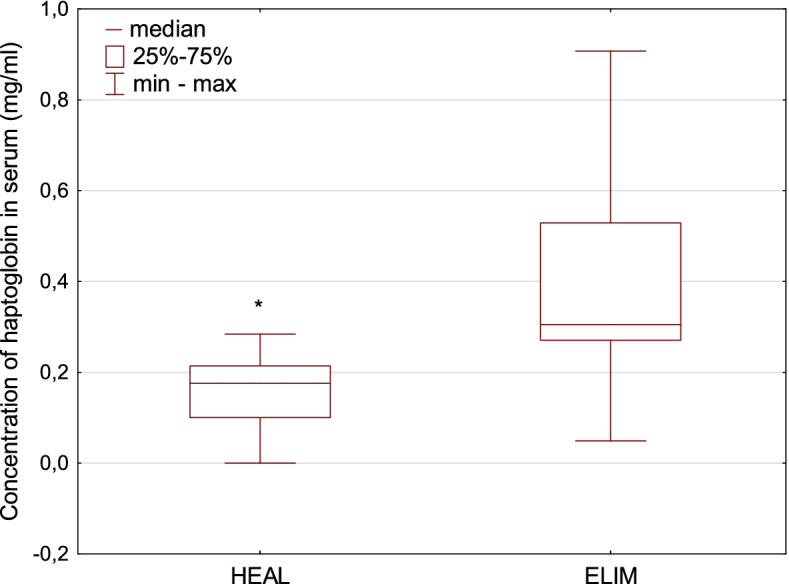
A boxplot for the concentration of haptoglobin in serum of European bison. The line inside the box is the median. The top and bottom lines of the box are the first and third quartiles, respectively. The top and bottom whiskers are the minimum and maximum concentrations. * - statistically significant differences between HEAL and ELIM (*p *< 0.01)

**Fig. 3:**
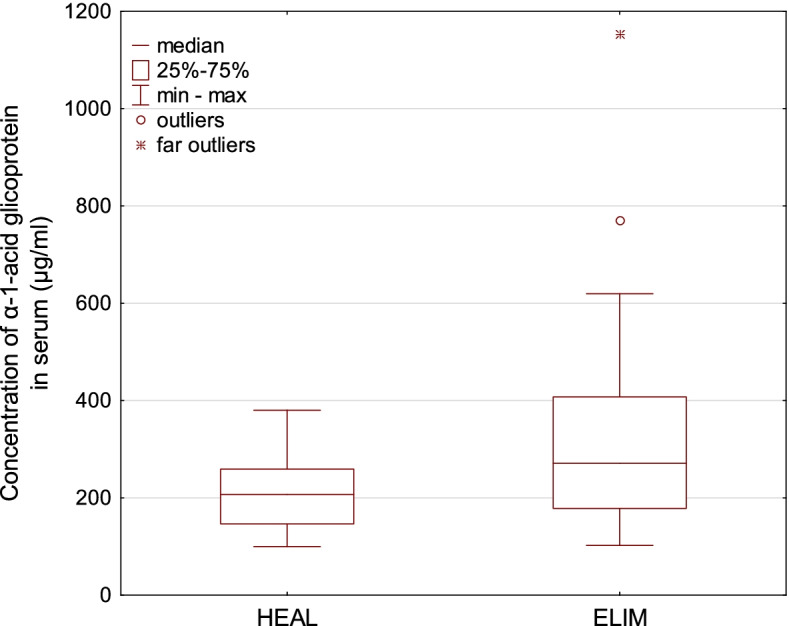
A boxplot for the concentration of α-1-acid glikoprotein in serum of European bison. The line inside the box is the median. The top and bottom lines of the box are the first and third quartiles, respectively. The top and bottom whiskers are the minimum and maximum concentrations, circles represent outliers, asterics represent far outliers

The median concentration of SAA in ELIM animals was almost 4 times higher than the median level in clinically healthy group. In ELIM, the median concentration was 70.81 µg/ml while in HEAL, the median concentration of SAA was 18.95 µg/ml. The interquartile range (IQR) equals 12.38-30.11 µg/ml for SAA concentration in HEAL, while in ELIM 50.50-112.46 µg/ml (Fig. 1). On other hand, the median concentration of Hp in HEAL group was over 2 times lower than in the eliminated animals with IQR equal to 0.1-0.214 mg/ml in HEAL and 0.270-0.592 mg/ml in ELIM. The median concentration of Hp in HEAL was 0.176 mg/ml, while in ELIM the median concentration of Hp was equal to 0.305 mg/ml (Fig. 2). The concentration of SAA and Hp was significantly higher in ELIM comparing to HEAL (*p* <0.01). Similar differences were also observed for AGP levels, however they were not statistically significant (*p* = 0.3950). The median levels of AGP in HEAL and ELIM were equal to 207.25 and 271.25 µg/ml, respectively. The IQR for AGP was 146.65-259.1 µg/ml in HEAL and 178.55-407.25 in ELIM µg/ml (Fig. 3).

## Discussion

In 2020, International Union for Conservation of Nature (IUCN) has changed European bison status from “Vulnerable” to “Near Threatened”, which proved that proper wildlife management is effective [26]. However, taking into account that modern European bison population was recreated from several individuals, continuous actions should be carried out to protect the species of the largest land European mammal. On-going health surveillance of European bison should be an important element of European bison protection strategy [2]. Our study suggest that determination of APPs might be a useful tool to assess health status of the individual or at the population level. In European bison, in the other study the most prevalent pathologies observed postmortally included: pneumonia, emphysema, nephritis, body traumas, posthitis/balanoposthitis in males and infestations of *Fasciola hepatica* and *Dictyocaulus viviparus* [27]. However, to our best knowledge there are no studies investigating APPs in European bison and their relation with different pathological conditions.

Regarding European bison`s immunology, there is only a report describing changes of immunoglobulins within the age [27]. Possibly, further studies may describe dynamics of APPs in different European bison disease. In the current preliminary study, we reported higher concentration of two (Hp, SAA) out of three investigated APPs in the eliminated due to poor health condition European bison. However, we cannot reject AGP as a potential marker for future studies using larger number of animals, since its concentrations were generally higher in animals from ELIM group, even though not statistically proven. The knowledge on usage of APPs as markers of physiological and pathological processes in wildlife species is lacking, even though they appear as desirable candidates for monitoring wildlife health and disease burden in the changing environment. Similar in the assumptions, the study of Glidden et al. [28] have demonstrated that Hp levels may be used to detect infections non-specifically and potential as a surveillance marker in African buffalo. Nevertheless, broaden experience may be drawn from cattle medicine, especially considering genetic relatedness among bovids. For example, Bagga et al. [14] have found APPs useful in diagnostics of lameness in cows, reporting SAA and Hp concentrations approximately 3 and 20, respectively times higher in lame cows comparing to non-lame cows. Similarly in our study the median concentration of SAA was almost 4 times higher in ELIM than in HEAL. The SAA levels obtained by Bagga in lame and non-lame cows (22.19 µg/ml and 8.89 µg/ml, respectively) are numerically lower that median SAA concentration in ELIM and HEAL (70.81 µg/ml and 18.95 µg/ml, respectively). Similarly the Hp concentrations in lame and non-lame cows (0.217 mg/ml and 0.012 mg/ml, respectively) are numerically lower than Hp concentration in ELIM and HEAL (0.305 mg/ml and 0.176 mg/ml, respectively. Dalanezi et al. [12] described increase in milk APPs in course of mastitis caused by different pathogens and suggested pathogen-specific APPs profiles. On the other hand, Moisa et al. [13] state that Hp concentration can be useful biomarker of respiratory diseases in dairy calves. The researcher reports that 0.195 mg/ml is the cut-off value for Hp as biomarker for bovine respiratory diseases. Comparing to our study, the European bisons from HEAL were below this value (0.176 mg/ml), while the animals from ELIM were above the cut-off point (0.305 mg/ml). Yet, Kęsik-Maliszewska et al. [29] have not proved differences in serum APPs excretion in experimentally infected with Schmallenberg virus calves, suggesting that not all infections induce measurable response. A team of Ansari-Lari et al. [30] analysed changes of Hp, Fb, SAA and albumin (Ab) levels in the course of post-traumatic reticulitis and peritonitis in cattle. Their study showed that the analysed proteins respond in a similar way and the particularly significant increase of their level was observed in acute diffuse peritonitis as compared to other forms of disease. Reduction of Ab level was observed in acute local peritonitis whereas serum concentration of Ab increased in a diffuse form of this condition. Moreover, APPs may be a useful indicators of the cattle welfare and stress [31, 32]. In the study by Saco et al. [31] serum levels of SAA and Hp in cattle kept under various environmental conditions were measured. They showed higher concentration of SAA in animals exposed to stress, whereas Hp values remained at similar level regardless of the animal maintenance. Comparing to our results, the SAA concentration both in ELIM and HEAL (70.81 µg/ml and 18.95 µg/ml, respectively) were significantly higher than cows kept under hardy conditions (3.46 µg/ml and 4.50 µg/ml). However, the levels of APPs may also vary in physiological conditions such as pregnancy, lactation [33, 34].

## Conclusions

To conclude, our preliminary study is the first report describing concentrations of the selected APPs (Hp, SAA and AGP) in European bison. Subsequently the study suggest that concentration of APPs may be used as a supportive tool, monitoring the health status (at individual and/or population level) and when making decisions regarding undesired individuals to be eliminated due to health reasons. The most significant limitations of our research was the moderate number of animals involved in the study. Further studies will include greater samples size in order to associate APPs levels with the most frequent pathologies of European bison from different populations, variable in exposure to pathogens or differences in herd management. Most importantly, the study confirmed the selection of animals for culling was diligent and thoughtful and could have been confirmed by APP up-regulation in sick animals.

## Data Availability

The datasets used and analysed in the current study are available from the corresponding author on reasonable request.

## Abbreviations

Ab: Albumins
AGP: α1-acid glycoprotein
APPs: Acute phase proteins
APR: Acute phase reaction
CVs: Coefficients of variation
ELIM: Individuals eliminated due to different pathological conditions
HEAL: Healthy individuals
Hp: Haptoglobin
IQR: Interquartile range
SAA: Serum amyloid A
SD: Standard deviation
